# VALIDATING THE CARE PREFERENCE ASSESSMENT OF SATISFACTION TOOL TO MEASURE QUALITY OF CARE IN NURSING HOMES

**DOI:** 10.1093/geroni/igz038.3241

**Published:** 2019-11-08

**Authors:** Caroline Madrigal, Kimberly VanHaitsma, Jacqueline Mogle, Donna Fick, Dennis Scanlon, Katherine M Abbott, Liza Behrens

**Affiliations:** 1 U.S. Department of Veterans Affairs, Providence, Rhode Island, United States; 2 The Pennsylvania State University, University Park, Pennsylvania, United States; 3 Miami University, Oxford, Ohio, United States; 4 University of Pennsylvania, Philadelphia, Pennsylvania, United States

## Abstract

The Institute for Healthcare Improvement’s Triple Aim calls for measures of the ‘patient care experience’ to understand and improve the quality of care delivery. But, quality measures in the nursing home (NH) historically lack the resident perspective. Measuring whether residents are satisfied with the fulfillment of their care preferences using the Care Preference Assessment of Satisfaction Tool (ComPASS) has been encouraged nationally by the Centers for Medicare and Medicaid (CMS); however, the ComPASS has not been validated as a measure of the resident care experience. The purpose of this study was to compare ComPASS to the Ohio NH Resident Satisfaction Survey (a widely accepted quality measure for reimbursement). We examined 196 resident responses from 28 NHs in Pennsylvania using multilevel modeling to account for dependencies in the data (residents in the same NH may respond similarly compared to residents from different NHs). Residents were 81.2 years old (SD= 11.1), female (70.4%), and white (80.1%). Residents with higher scores on the ComPASS reported significantly higher levels of satisfaction with care (B=2.94, SE B=0.59, p<0.000). Results from this study support the potential use of ComPASS to measure, track, and improve the quality of NH care. Using ComPASS aligns with CMS’s Section F of the Minimum Dataset, an assessment of residents’ preferences which promotes the delivery of more person-centered care. Ultimately, ComPASS can help benchmark the quality of the resident care experience across facilities which aids staff, facilities, policy-makers, and NH-shoppers in improving decision-making and care delivery.

